# Novel *rpsK* / *rpsD* primer-probe assay improves detection of *Campylobacter jejuni* and *Campylobacter coli* in human stool

**DOI:** 10.1371/journal.pntd.0012018

**Published:** 2024-03-01

**Authors:** Francesca Schiaffino, Craig T. Parker, Paul F. Garcia Bardales, Steven Huynh, Katia Manzanares Villanueva, Evangelos Mourkas, Ben Pascoe, Pablo Peñataro Yori, Maribel Paredes Olortegui, Eric R. Houpt, Jie Liu, Kerry K. Cooper, Margaret N. Kosek

**Affiliations:** 1 Faculty of Veterinary Medicine, Universidad Peruana Cayetano Heredia, San Martin de Porres, Lima, Peru; 2 Division of Infectious Diseases and International Health, School of Medicine, University of Virginia, Charlottesville, Virginia, United States of America; 3 Agricultural Research Service, U.S. Department of Agriculture, Produce Safety and Microbiology Research Unit, Albany, California, United States of America; 4 Asociacion Benefica Prisma, Iquitos, Peru; 5 Ineos Oxford Institute for Antimicrobial Research, Department of Biology, University of Oxford, Oxford, United Kingdom; 6 Centre for Genomic Pathogen Surveillance, Big Data Institute, University of Oxford, Oxford, United Kingdom; 7 School of Public Health, Qingdao University, Qingdao, China; 8 School of Animal and Comparative Biomedical Sciences, University of Arizona, Tucson, Arizona, United States of America; 9 The BIO5 Institute, University of Arizona, Tucson, Arizona, United States of America; Mahidol Univ, Fac Trop Med, THAILAND

## Abstract

*Campylobacter* causes bacterial enteritis, dysentery, and growth faltering in children in low- and middle-income countries (LMICs). *Campylobacter* spp. are fastidious organisms, and their detection often relies on culture independent diagnostic technologies, especially in LMICs. *Campylobacter jejuni* and *Campylobacter coli* are most often the infectious agents and in high income settings together account for 95% of *Campylobacter* infections. Several other *Campylobacter* species have been detected in LMIC children at an increased prevalence relative to high income settings. After doing extensive whole genome sequencing of isolates of *C*. *jejuni* and *C*. *coli* in Peru, we observed heterogeneity in the binding sites for the main species-specific PCR assay *(cadF) and* designed an alternative *rpsKD-*based qPCR assay to detect both *C*. *jejuni* and *C*. *coli*. The *rpsKD-*based qPCR assay identified 23% more *C*.*jejuni/ C*.*coli* samples than the *cadF* assay among 47 *Campylobacter* genus positive *cadF* negative samples verified to have *C*. *jejuni* and or *C*. *coli* with shotgun metagenomics. This assay can be expected to be useful in diagnostic studies of enteric infectious diseases and be useful in revising the attribution estimates of *Campylobacter* in LMICs.

## Author summary

*Campylobacter* is a leading cause of gastroenteritis among children living in resource poor settings. Infections are predominantly caused by *Campylobacter jejuni* and *Campylobacter coli*. To estimate the burden of *Campylobacter*, nucleic acid diagnostic testing has been utilized in large population based epidemiologic studies. However, there is evidence of heterogeneity in the binding sites for the main *C*. *jejuni* and *C*. *coli* qPCR assay utilized. This study presents and validates an alternative *rpsKD*-based qPCR assay to detect both *C*. *jejuni* and *C*. *coli*. Improved detection of these two species is expected to impact diagnostic studies of enteric infectious disease and improve estimates of the overall burden of *Campylobacter*.

## Introduction

*Campylobacter* has been classified as a leading cause of bacterial gastroenteritis and growth stunting in children living in poverty. *Campylobacter jejuni* and *Campylobacter coli* are the predominant *Campylobacter* species associated with gastroenteritis, both in pediatric populations and in cases of travellers’ diarrhea [1–3]. Additionally, *C*. *jejuni*, *C*. *coli* and other *Campylobacter* spp. are detected from infants with asymptomatic *Campylobacter* infections in low- and middle-income countries (LMICs) where *Campylobacter* infections are endemic [4,5] and are associated with growth deficits even when they are asymptomatic [6]. The high prevalence of this pathogen globally has been supported by large scale population-based studies that have used nucleic acid-based diagnostics to attribute cases of diarrhoea to this pathogen [7–11].

The performance of culture independent diagnostic technologies (CIDTs), such as quantitative polymerase chain reaction (qPCR), also known as real-time PCR, is particularly important for the detection of *Campylobacter* spp. in LMICs. *Campylobacter* spp. are fastidious microorganisms that are challenging to culture and isolate requiring a variety of specific atmospheric and nutritional requirements that are often not easily obtained in LMICs [12]. Moreover, nucleic acid based diagnostic tests have been shown to detect *Campylobacter* spp. at levels lower than those required for culture based isolation [13]. The target for *Campylobacter* genus detection is usually the 16S rRNA gene [4,5,13,14] or the chaperonin protein cpn60 [1,15,16] that has been used to detect both common thermotolerant *Campylobacters*, such as *C*. *jejuni* and *C*. *coli* and less common thermotolerant and non-thermotolerant *Campylobacter* species, such as *C*. *upsaliensis*, *C*. *concisus* and “*Candidatus* Campylobacter infans.” A variety of genetic targets have been utilized to detect *C*. *jejuni* and *C*. *coli*. Assay targets may be specific for either species, *mapA* and *hipO* for *C*. *jejuni*, *ceuE* and *glyA* for *C*. *coli* [17,18]. Alternatively, a *cadF* primer-probe set was designed to detect both *C*. *jejuni* and *C*. *coli* [19]. In large scale studies, a combination of the chaperonin protein cpn60 and *cadF* primer-probe sets have been used in TaqMan Array cards for simultaneously detecting *Campylobacter* spp. and *C*. *jejuni*/ *C*. *coli*, respectively [8,20]. For samples that tested positive for *Campylobacter* spp. and *C*. *jejuni*/ *C*. *coli*, the samples were scored as possessing *C*. *jejuni*/ *C*. *coli* and those that tested positive for *Campylobacter* spp. and negative for *C*. *jejuni*/ *C*. *coli* were scored as non-*C*. *jejuni*/ C. *coli* [12,21].

Several studies examining fecal samples from infants in LMICs detected non-*C*. *jejuni*/ C. *coli Campylobacter* at levels as high as 70% of *Campylobacter* infections, leaving an unacceptably large group of *Campylobacter* species undesignated at the species level [12,21]. Recently, shotgun metagenome sequencing of stool samples that tested qPCR positive for *Campylobacter* spp. and negative for *C*. *jejuni*/ *C*. *coli* (*cadF*) demonstrated that nearly 64% of the samples possessed either *C*. *jejuni* or *C*. *coli*, and sometimes both [5]. This major underestimation of the overall prevalence of *C*. *jejuni* and *C*. *coli* in fecal samples suggested that an improved primer-probe set for the detection of *C*. *jejuni*/ *C*. *coli* was needed.

A community-based cohort study near Iquitos, Loreto, Peru provided fecal samples from asymptomatic and diarrheal samples to test an alternative *rpsKD-*based qPCR assay to detect both *C*. *jejuni* and *C*. *coli*. Here we report the development and assessment of this assay that would have an improved sensitivity relative to *cadF* for the detection of *C*. *jejuni* and *C*. *coli*.

## Materials and methods

### Ethics statement

Human fecal samples used in this study are part of a study approved by the Institutional Review Board of Asociacion Benefica Prisma (Lima, Peru) and the University of Virginia (Charlottesville, VA, United States). Written consent to participate in the study was obtained from the parents or legal guardians of children. Participants of both studies consented for further use of biological specimens.

### Biological samples

Archived fecal samples were derived from children enrolled in a community-based cohort study in Iquitos, Loreto, Peru. The study initiated in 2021 and is currently ongoing. Children are enrolled within 17 days of birth and followed for the first two years of life. Children were visited twice weekly to create a continuous daily record of early life childhood illness. Fecal samples are collected monthly as well as each time a child has diarrhea (defined as >3 unformed stools in a 24-hour period). This study included randomly selected fecal samples available during the first 12 months of the study. Archived fecal samples had been stored at -80°C after initial collection.

### Development of *rpsK*/*rpsD* primer-probe assay

The development of a *C*. *jejuni* / *C*. *coli* TaqMan based primer-probe to replace the *cadF* primer-probe was initiated using 12 whole genome sequences consisting of eight *C*. *jejuni* and four *C*. *coli* genomes. Genomic loci that were >90% identical between the two species were examined in detail. Among regions identified, was a locus of 2,808 nucleotides (nts) that included *rpsM*-*rpsK*-*rpsD-rpoA-rplQ*, a genome region encoding the 30S ribosomal protein. This region from the 12 genome sequences was aligned using MAFFT [22] within Geneious Prime (v2023.2.1; Biomatters, Ltd., Auckland, New Zealand) and a consensus sequence was utilized to identify forward primer, reverse primer and probe sequences of 100% identity between the genomes using Primer3 tool (v2.3.7) [23] within Geneious Prime.

### Validation and evaluation of *rpsK*/*rpsD* primer-probe assay

#### In silico validation

The selected sequences for the *rpsK*/*rpsD* primers and probe (**Table 1**) were compared against 9,000 *C*. *jejuni* and *C*. *coli* genomes in the pubMLST database (https://pubmlst.org/organisms/campylobacter-jejunicoli) using BLASTN. To ensure that other closely related species are not amplified, we examined by BLASTN against 300 *C*. *upsaliensis* and 300 *C*. *lari* genomes from the same database.

**Table 1:** Primer and probes used to detect *Campylobacter* spp. and *Campylobacter jejuni* / *Campylobacter coli*.

Target	Nombre	Sequence	Source
*Campylobacter* genus (16S rRNA)	16s_Fw	5’- CAC GTG CTA CAA TGG CAT AT -3	[12]
	16s_Rv	5’- GGC TTC ATG CTC TCG AGT T -3’	
	16s_Probe	5’- /56-FAM/CAG AGA ACA /ZEN/ ATC CGA ACT GGG ACA /3IABkFQ/ -3’	
*Campylobacter coli* and *Campylobacter jejuni (cadF)*	cadF_Fw	5’- CTG CTA AAC CAT AGA AAT AAA ATT TCT CAC -3’	[12]
	cadF_Rv	5’- CTT TGA AGG TAA TTT AGA TAT GGA TAA TCG -3’	
	cadF_Probe	5’ -/56-VIC/CAT TTT GAC /ZEN/ GAT TTT TGG CTT GA/3IABkFQ/ -3’	
*Campylobacter coli* and *Campylobacter jejuni (rpsK/rpsD)*	Rps_Fw	5’- AGG TGC TAT GGA AGG AAT CAA AGT -3’	This study
	Rps_Rv	5’- TAA TGC TAAG CTA ACA CCA AAG CG -3’	
	Rps_Probe	5’ -/56-VIC/ CCT AAG CGT CGT CGT GTC TAA GAT /3IABkFQ/ -3’	

#### In vitro evaluation

The performance of the new *rpsK*/*rpsD* primer-probe assay was evaluated with a set of archived (-70°C) stool samples from children under 24 months of age. All samples were processed using both assay **[A]** which consisted of the Taqman based multiplex assay to detect *Campylobacter* spp. (16S rRNA gene) and *Campylobacter jejuni* / *Campylobacter coli* using the *cadF* gene and assay **[B]** which consisted of the Taqman based multiplex assay to detect *Campylobacter* spp. (16S rRNA gene) and *C*. *jejuni* / *C*. *coli* using the newly validated *rpsK*/*rpsD* gene fragment. Primer and probe sequences of both assays is presented in **Table 1.**

Fecal DNA was extracted from 0.2 grams of feces using the QIAamp DNA Stool Mini Kit (Qiagen, Carlsbald, CA), according to the manufacturer’s instructions. A negative control consisting of RNA and DNA free water was used for each extraction set. The final assay consisted of a 25 μL final reaction mixture with 12.5 μL of Environmental Master Mix (2X) (Applied Biosystems, Foster City, CA), forward and reverse primers (0.2 μM), probes (0.1 μM), 1 μL of DNA template and RNase and DNase free water (Ambion, Thermo Fisher Scientific, Waltham, MA, USA). Both qPCR assays were performed on a QuantStudio 7 Flex (Applied Biosystems, Foster City, CA) using the following cycling conditions: 95°C for 10 minutes followed by 45 cycles of 95°C for 15 seconds and 60°C for 1 minutes. Custom manufactured double-stranded synthetic DNA fragments (gBlocks, Integrated DNA Technologies, Coralville, IA, USA) were used as positive controls. Negative template controls (RNase and DNase free water) were included in each amplification reaction.

Standard curves for each marker were prepared using 10-fold serial dilutions of synthetic positive controls (6.0 x 104–6.0 x 10^0^) gene copies/μL. For both assays, a cut-off cycle threshold of 38 was used to determine positivity.

#### Shotgun metagenomic DNA sequencing

Fecal samples that were positive for *Campylobacter* spp. (16S rRNA gene), but negative for *C*. *jejuni* and *C*. *coli* (either with the *cadF* primer set or with the *rpsK*/*rpsD* primer set) underwent shotgun metagenomic sequencing. DNA sequencing libraries were prepared using Illumina DNA Prep Tagmentation kit (Illumina, San Diego, CA), following the manufacturer’s instructions except for the following changes. The insert size was increased to a range of ~375–1100 bp by reducing the 1st and 2nd volumes of Sample Purification Beads to 40 μl and 11 μl, respectively. This modification resulted in larger inserts compared to the mostly below 300 bp inserts obtained using the manufacturer’s protocol. The final elution volume of the libraries was in 10 μl of Illumina resuspension buffer. Illumina-DNA/RNA UD Indexes Plate A, B, C and D dual index adapters were ordered from Integrated DNA Technologies (Coralville, IA) and used at 1 μM final concentration. Instead of pooling equal volumes, individual libraries were quantified using the KAPA Library Quantification Kit (Roche), since we found qPCR to be a more accurate quantification than using equal volume. Libraries were quantified in 10 μl volume reactions and 90-s annealing/extension PCR, and then pooled and normalized to 4nM. Pooled libraries were re-quantified by ddPCR on a QX200 system (Bio-Rad, Hercules, CA), using the Illumina TruSeq ddPCR Library Quantification Kit and following the manufacturer’s protocols. Libraries were sequenced using a MiSeq Reagent Kit v2 (500-cycles) on a MiSeq instrument (Illumina) at 16 pM, following the manufacturer’s protocols. Average sequence read lengths for each sample was 200 nucleotides. Genomes were assembled using SPAdes assembler 3.15.5 within Geneious Prime 2023.1.2 (Biomatters, Ltd., Auckland, New Zealand). Short read sequence data for all the samples are available at NCBI’s Sequence Read Archive (SRA) and are associated with BioProject Accession PRJNA834762.

## Results

### In silico validation

To increase the efficacy of the *C*. *jejuni/C*. *coli* qPCR, we endeavored to replace the *cadF* primers/probe set that failed to identify *C*. *jejuni* and *C*. *coli* in positive stool samples [5]. First, we identified genomic regions present in *C*. *jejuni* and *C*. *coli* possessing synteny and greater than 90% sequence identity, including *rpsM*-*rpsK*-*rpsD-rpoA-rplQ*, a genome sequence encoding small ribosomal proteins. Second, we designed the *rpsK*/*rpsD* primers and probe set from the *rpsM*-*rpsK*-*rpsD-rpoA-rplQ* consensus sequence from aligned genomes of eight *C*. *jejuni* and four *C*. *coli* strains (**Table 1**). Next, we compared the primers and probe against 9,000 *C*. *jejuni* and *C*. *coli* genomes in the pubMLST database by BLASTN with over 96% of the genomes having 100% identity to the set. We also compared the primers and probe to the other principal thermotolerant *Campylobacters*, *C*. *lari* and *C*. *upsaliensis* to predict cross reactivity. When the primer probe set was compared to 300 *C*. *lari* genomes, we determined that the forward primer had only 2 differences out of 24 nts, but the reverse primer contained 8 differences out of 24 nts, and the probe had 4 differences out of 24 nts. Compared against 300 genomes of *C*. *upsaliensis*, the forward primer had 5 differences out of 24 nts, the reverse primer contained 5 differences out of 24 nts and the probe had 6 differences out of 24 nts and thus cross reactivity of the primer probe mix with these species was deemed highly unlikely.

### Evaluation of cohort samples

A total of 242 human fecal samples were assayed for both *Campylobacter* spp. and *C*. *jejuni/C*. *coli* by qPCR. In all assays, presence of *Campylobacter* spp. was determined using a 16S rRNA primer/probe set. The presence of *C*. *jejuni* and/or *C*. *coli* was determined using *C*. *jejuni/C*. *coli cadF* primer-probe assay [19] or *rpsK*/*rpsD* primer-probe assay designed in this study. **Table 2A** shows results obtained after running all 242 samples with *Campylobacter* 16S rRNA gene and the *cadF* gene primer-probe assay. *Campylobacter* spp. was detected in 19.4% (47/242) of samples, and *C*. *jejuni*/ *C*. *coli* in 6.6% (16/242) of samples using the *cadF* assay. **Table 2B** shows results obtained by running the same samples with the *Campylobacter* 16S rRNA primer-probe assay and *rpsK*/*rpsD* gene primer-probe assay. In this case, *Campylobacter* spp. was detected in 19.0% (46/242) of samples, and *C*. *jejuni* and/or *C*. *coli* in 11.2% (27/242) of samples. In other words, the *cadF* assay identified 34.0% (16/47) of *Campylobacter* spp. positive samples as *C*. *jejuni*/ *C*. *coli*, whereas the *rpsK*/*rpsD* assay identified 58.7% (27/46) of *Campylobacter* spp. positive samples as *C*. *jejuni*/ *C*. *coli*, increasing the attribution of *Campylobacter* associated diarrheal to *C*. *jejuni* and *C*. *coli* by over 20%.

**Table 2:** Contingency Tables of *Campylobacter* spp (16S rRNA), and *Campylobacter jejuni*/ *Campylobacter coli* using the cadF primer probe set and the *rpsK/rpsD* primer probe set. Using the *cadF* primers, 66% (31/47) *Campylobacter* infections would be assigned to “other Campylobacter” whereas only 41.3% (19/46) *Campylobacter* infections would be assigned to this category using newly designed *rpsK/rpsD* primer set. No specimens that were *cadF* positive were negative using *rpsK/rpsD* primers.

**A. Contingency table of *Campylobacter* spp. (16S rRNA) and *Campylobacter jejuni*/ *Campylobacter coli* detection using the *cadF* primer probe set.**			
**qPCR Target**	*C*. *jejuni*/ *C*. *coli* (cadF)		
*Campylobacter* spp. (16S)	Positive	Negative	Total
Positive	16	31	47
Negative	0	195	195
Total	16	226	242
**B. Contingency table of *Campylobacter* spp. (16S rRNA) and *Campylobacter jejuni*/ *Campylobacter coli* detection using the *rpsK/rpsD* primer probe set.**			
**qPCR Target**	*C*. *jejuni*/ *C*. *coli* (*rpsK/rpsD*)		
*Campylobacter* spp. (16S)	Positive	Negative	Total
Positive	27	19	46
Negative	0	196	196
Total	27	215	242
**C. Contingency table of *Campylobacter jejuni*/ *Campylobacter coli* detection using the cadF primer probe set and the *rpsK/rpsD* primer probe set.**			
**qPCR Target**	*C*. *jejuni*/ *C*. *coli* (cadF)		
*C*. *jejuni*/ *C*. *coli* (*rpsK/rpsD*)	Positive	Negative	Total
Positive	16	11	27
Negative	0	215	215
Total	16	226	242

When comparing the performance of the *cadF* primer and the *rpsK*/*rpsD* primer in a single contingency table (**Table 2C**), we observe that 40.7% (11/27) of *rpsK*/*rpsD* positive samples were classified as negative with the *cadF* primer, while none of the *cadF* positive samples were classified as negative by the *rpsK*/*rpsD* primer.

### Assessment of qPCR results

To assess the detection accuracy of the two *C*. *jejuni*/ *C*. *coli* qPCR assays, we performed shotgun metagenomic sequencing of stool samples. Of the 31 samples that were *Campylobacter* spp. positive for the 16S rRNA gene target and *cadF* negative, shotgun metagenomic DNA sequencing identified *Campylobacter jejuni* and/or *Campylobacter coli* reads in 10 samples, thus demonstrating a false negativity of 33.3% (10/31) of *cadF* in the identification of *C*. *jejuni* and *C*. *coli* in patient samples **(Table 3)**. In respect to species of *Campylobacter* other than *C*. *jejuni* and *C*. *coli*, *C*. *concisus* was identified in 8 samples (in 5 cases as the only *Campylobacter* species identified, and once each with *C*. *coli*, *C*. *hominis*, or *C*. *concisus*, respectively). *C*. *hominis* was identified only in one sample, *C*. *infans* in four samples (once with C. *concisus*, once with C. upsaliensis and once as the only *Campylobacter* species identified) and *C*. *upsaliensis* in a single sample. Two samples had reads that matched to more than one *Campylobacter* spp., and 9 samples had no reads that match to any *Campylobacter* spp. Of these 9 samples, Ct values for 16S rRNA primer-probe set were higher than 37.0. Finally, there were not enough reads for an appropriate analysis to be performed in three samples.

**Table 3:** Number of reads detected for *Campylobacter jejuni*, *C*. *coli*, *C*. *upsaliensis*, and *C*. *helveticus*, *C*. *concisus* and *C*. *infans* in human fecal samples, and qPCR status. Samples highlighted in green show highlight 8 of 11 samples that were negative using *cadF* primers, positive with new *rpsK/rpsD* primers, and confirmed as having *C jejuni* or *C*. *coli* infection by metagenomic analysis.

Sample ID	Primer Set A (Ct value)		Primer Set B (Ct value)		qPCR Interpretation			Metagenomic Interpretation		Metagenomic Ouput						
	*Campylobacter* spp.	*C*. *jejuni/ C*. *coli*	*Campylobacter* spp.	*C*. *jejuni/ C*. *coli*	cadF negative and *rpsK/rpsD* positive	16S rRNA positive & *rpsK/rpsD* negative	16S rRNA positive & *cadF* negative	*C*. *jejuni/ C*. *coli* positive	Other *Campylobacter* spp. Positive	Total Number of Reads	*C*. *jejuni*	*C*. *coli*	*C*. *upsaliensis*	*C*. *concisus*	*C*. *hominis*	*C*. *infans*
12881	27.6		28.3	31.7	x		x	x		2,162,660	57	5				
13506	35.2		34.3	38.0	x		x	x		1,909,830	8					
13755	26.2		25.2	28.4	x		x	x		1,526,474	4	30				
13763	36.6	43.2	33.5	36.9	x		x	x	x	895,208	6	7		67	2	
15073	33.4		31.3	33.4	x		x	x		1,474,996	2	13				
15460	32.1		31.6	34.5	x		x	x		1,857,668		4				
15869	32.8		32.3	34.5	x		x	x		1,352,160	2					
16171	32.4	38.0	32.0	34.8	x		x	x		1,454,002	2	6				
13759	36.7	40.3	36.5	37.6	x		x		x	1,121,504				8	2	
13767			36.6	37.6	x					105,296						
15450	35.9		33.5	36.8	x		x			2,817,542						
15867	23.7		23.5			x	x		x	231,234			2			24
15356	24.6		22.7			x	x		x	2,611,646				2		28
17462	29.8		29.2			x	x		x	190,138						34
16786	29.8		28.5			x	x		x	453,644						14
15656	34.4	38.7	33.6	38.1		x	x		x	1,464,530				4		
15660	35.4		34.4	38.2		x	x	x	x	2,066,290	2	6				
14439	35.8	40.5	34.5	38.8		x	x		x	847,536					164	
13773	35.9	41.5	36.3	38.5		x	x	x		2,859,766	2					
14465	36.7		37.9			x	x		x	761,546				8		
16437	36.9		35.6	39.5		x	x		x	1,250,708				2		
13771	37.1	40.0	34.5			x	x	x		3,450,952	7	3				
16165	37.4						x			3,649,528						
16179	37.5		39.5				x			1,742,920						
13761	37.7						x		x	3,414,758				22		
17515	37.7		38.9				x			2,036,728						
16175	37.8	41.4	38.5				x			435,602						
15456	37.9						x			2,431,290						
13242	37.9						x			249,728						
13276	37.9						x			1,706,912						
15663	38.0						x		x	198,706				6		
13240	39.2		**37.3**			x				950,806						
13070			37.6			x				1,675,884						
14826			37.4			x				629,710						
15526		40.0	37.6			x				1,652,978						
16784			37.9			x				2,211,672						
12991	33.6		34.5	39.4		x	x			To few reads						
13753			37.6			x				To few reads						
15075	38.1		34.8			x				To few reads						
Three samples, 12991, 13753 and 15075 had too few reads for a proper analysis.																
Samples highlighted in green demonstrate improved performance of Primer Set B Ct = Cycle Threshold																

Of the 19 samples that were *Campylobacter* 16S rRNA positive and *rpsK*/*rpsD* negative metagenomic analysis identified *C*. *jejuni* and *C*. *coli* in only two samples for a false negativity of 11% (2/19). These two samples had very few reads for *C*. *jejuni* and *C*. *coli*, and both had a Ct value over 34 in the *rpsK/rpsD* qPCR assay and 16S rRNA Ct values of 37.3 and 35.4. Finally, of the 11 samples that were *cadF* negative, yet *rpsK*/*rpsD* positive, *C*. *jejuni* reads and/or *C*. *coli* were identified by the *rpsK*/*rpsD* primer set in 8 of the 11 samples. Of the 3 remaining samples, the Ct obtained by the *rpsK*/*rpsD* primer set was higher than 36.5. Details of the total number of reads and reads matched to specific *Campylobacter* spp. species are shown in **Table 3.**

## Discussion

Molecular diagnostics has greatly advanced our understanding of the epidemiology of enteric diseases. However, for certain species, such as *Campylobacter*, the underrepresentation of whole genomes or extensive genomic data from different geographic areas impairs the development of effective primers and probes. After demonstrating the inadequate performance of the *cadF* assay [5], we developed a new qPCR assay for the detection *C*. *jejuni*/ *C*. *coli* based on *rpsK*/*rpsD* that reduced the false negative detection results observed with a *cadF-*based qPCR assay. Overall, we detected *Campylobacter* spp. in 19% (47/242) of fecal samples from infants under one year that are enrolled in a cohort study in Amazonian Peru. Among these 47 samples the *cadF* qPCR assay detected *C*. *jejuni*/ *C*. *coli* in only 16 samples while the *rpsK*/*rpsD* qPCR assay detected *C*. *jejuni*/ *C*. *coli* in 27, a 23% (11/47) increase in detection of attribution of *Campylobacter* species to *C*. *jejuni/ C*. *coli*. Furthermore, we validated *C*. *jejuni* and/ or *C*. *coli* in 8/11 of the samples with discordant *cadF* and *rpsK/rpsD* results using shotgun metagenomic sequencing. The three samples that lack *C*. *jejuni* and/or *C*. *coli* sequencing reads are not necessarily indicative of the *rpsK*/*rpsD* qPCR providing false negative results. Shotgun metagenomic sequencing does not include any target amplification steps and positive results not only depend on the presence of *C*. *jejuni* and/or *C*. *coli* but also the levels of other bacterial species in the fecal microbiome that essentially saturate the available reads with non-informative data. Adding a pre-enrichment amplification of broadly cross-reactive primers that amplify across the highly diverse *Campylobacter* genus may in the future address this technical issue. Here, the shotgun metagenomic sequencing identified the presence of *Campylobacter* spp. other than *C*. *jejuni*/ *C*. *coli* including *C*. *concisus* and “*Candidatus* Campylobacter infans” at a prevalence high enough to serve as a reminder that all *Campylobacter* species from clinical specimens should not be assumed to be *C*. *jejuni* or *C*. *coli*. In this study, even with improved primers for the detection of *C*. *coli/C*. *jejuni* we found that over 40% of the *Campylobacter* identified by the 16S RNA gene target were not clearly assigned to *C*. *jejuni/ C*. *coli* by qPCR. This remains an important diagnostic gap. The prevalence of *Campylobacter* genus positivity was 19% in this population, but only 11.1% were positive to *C*. *jejuni/ C*. *coli* with the newly developed improved primer-probe set described here.

The clinical significance of other Campylobacter species, including ’Candidatus Campylobacter infans’, *Campylobacter upsaliensis*, and *Campylobacter concisus*, in relation to the burden of diarrhea in early childhood and its subsequent consequences, requires further investigation through large-scale population-based studies. The development of a multiplex qPCR primer-probe assay capable of detecting other *Campylobacter* species would be beneficial. The primers described in this article are intended to improve the targeted detection of *C*. *jejuni* and *C*. *coli*, which are commonly recognized as the most medically important species of Campylobacter present globally. The major limitation of this assay and molecular diagnostics in general is that the diagnostic capacity of hospitals and other health care settings in low and middle-income countries limits the utilization of this primer-probe assay in clinical settings in areas where they are most needed. However, improved diagnostics in the research setting and in large epidemiologic studies still allows improved molecular diagnostics to inform disease burden estimates and prioritize the development of disease specific interventions.

We have previously shown that the detection of *C*. *jejuni/ C*. *coli* using the single gene target *cadF* is not optimal [4,5]. Specifically, we demonstrated that within fecal samples that were positive for *Campylobacter* spp. but negative for *C*. *jejuni*/ *C*. *coli* using the *cadF* gene target, over 50% of the samples possessed *C*. *jejuni* and *C*. *coli* sequence reads by shotgun metagenomic sequencing [4,5]. Interestingly, the number of *Campylobacter* sequencing reads in this current study was lower than the previous studies. In this study, the number of reads range from 2 to 57 while in the previous studies [4,5], the number of reads ranged from 2 to >7000 for *C*. *jejuni*/ *C*. *coli*. Similarly for non-*C*. *jejuni*/*C*. *coli* reads, the difference was even wider with reads ranging from 2 to 164 in this study compared to a range of 2 to approximately 70,000 reads [4].

There are a number of reasons that the current samples provided fewer *Campylobacter* reads. First, the samples from the previous study were all from infants that had medically attended diarrhea while the stools in this in this study were from symptomatic and asymptomatic infants in a cohort study. Second, the age of the infants was distinct, with the previous studies under 2 versus under 1 year old in this study. Finally on our previous study we utilized a different sequencing protocol using KAPA library preparation. This method creates libraries through mechanical shearing, creating greater diversity of reads regarding DNA GC content. In this study, we used Illumina library kits that rely on enzymatic cleavage, which give lower coverage for DNA with low GC content. This led to the hypothesis that the Illumina library kit method would reduce the number of reads from low GC content organisms, such as Campylobacters, during microbiome library construction. However, we repeated the extraction method using KAPA library preparation, obtaining similar read values. and thus, proving similar performance of both library preparation methods.

Although other qPCR assays have been developed to detect *C*. *jejuni* and *C*. *coli* separately such as *hipO* for *C*. *jejuni* and 23S rRNA for *C*. *coli* [24,25], these targets have failed to demonstrate high reproducibility [26]. For this reason, the *cadF* gene target, which detects both *C*. *jejuni* and *C*. *coli* was adopted as a more relevant target, as both species produce similar clinical syndromes. There are important implications in the underperformance of the most common target for the detection of the two most medically important species of *Campylobacter*. Global estimates of disease, particularly those of low-income countries, have relied on *cadF* diagnostics for causal attribution, and thus disease burdens have been systematically underestimated for *C*. *jejuni* and *C*. coli one of the principal etiologies of bacterial enteritis globally and a vaccine target [8,15,20]. Nucleic acid diagnostics improves the sensitivity of *Campylobacter* diagnostics compared to culture dependent methods. This work demonstrates improved performance of the *rpsK/rpsD* target for the identification of *C*. *jejuni* and *C*. *coli* and demonstrates the utility of metagenomics in validating the assay performance as a useful and practical strategy in assessing the performance of assays of fastidious organisms such as *Campylobacter*.
